# Contrasting seasonal patterns of telomere dynamics in response to environmental conditions in the ectothermic sand lizard, *Lacerta agilis*

**DOI:** 10.1038/s41598-019-57084-5

**Published:** 2020-01-13

**Authors:** Jannike Axelsson, Erik Wapstra, Emily Miller, Nicky Rollings, Mats Olsson

**Affiliations:** 1 Department of Biological and Environmental Sciences, University of Gothenburg, Gothenburg, Sweden; 2 School of Natural Sciences, University of Tasmania, Hobart, Tas 7001 Australia; 3 School of Life and Environmental Sciences, The University of Sydney, Sydney, NSW 2006 Australia

**Keywords:** Ecology, Ecology

## Abstract

Telomeres, the protective, terminal parts of the chromosomes erode during cell division and as a result of oxidative damage by reactive oxygen species (ROS). Ectotherms rely on the ambient temperature for maintaining temperature-dependent metabolic rate, regulated through behavioural thermoregulation. Their temperature-dependant metabolism, hence also the ROS production, is indirectly regulated through thermoregulation. Consequently, a potential causal chain affecting telomere length and attrition is: temperature (in particular, its deviation from a species-specific optimum) – metabolism - ROS production – anti-oxidation - telomere erosion. We measured telomere length in sand lizards (*Lacerta agilis*) using qPCR on blood samples from 1998–2006. Effects of climatological parameters (mean temperature and average sunshine hours) in the summer and winter preceding telomere sampling were used as predictors of telomere length in mixed model analysis. During the lizards’ active period (summer), there was a largely negative effect of mean temperature and sun on telomere length, whereas a combined measure of age and size (head length) was positively related to telomere length. During the inactive period of lizards (winter), the results were largely the opposite with a positive relationship between temperature and sunshine hours and telomere length. In all four cases, thermal and age effects on telomere length appeared to be non-linear in the two sexes and seasons, with complex response surface effects on telomere length from combined age and thermal effects.

## Introduction

Telomeres are the terminal parts of the chromosomes with simple tandem repeats ending as a single-stranded t-loop^1^. They keep the chromosomes intact, protecting them from degradation and end-to-end fusion^2^. Telomeres shorten during replication and may reach an unstable, critical point, which induces cell arrest and apoptosis^3^. Aerobic metabolism generates highly reactive and cell-damaging reactive oxygen species (ROS)^4,5^, to which G-rich telomeres are particularly sensitive^6,7^. Thermal shifts restrain the oxygen delivery system and ROS production increases during heat stress, hence telomere erosion is elevated by a rise in temperature, at least in some taxa^8^. The loss of telomeric bases is counterbalanced by telomerase, which elongates telomeres with its RNA template^2^, in species-specific -, chromosomal location-specific -, and chromosomal- and telomere length-specific manners (e.g.^9,10^,).

Recent years has seen an increased focus on telomere dynamics in natural populations with a focus on links to life history, reproduction, ageing trajectories and survival (e.g.^9–13^,). Specifically, there is much interest in the relationship between environmental conditions and telomere dynamics and their long- and short-term effects in a range of taxa, but thus far ectotherms have been poorly represented in the literature^9^. As ectotherms, reptiles rely on environmental conditions for maintaining their body temperature and regulate this via behavioural thermoregulation^14–16^. Over short time periods (e.g., daily) this is achieved by, for example, shuttling heliothermy, burrowing, and altered activity patterns. Over longer time scales, aestivation or hibernation modifies the relationship between environmental conditions and body temperature. A consequence of ectothermy is that the metabolic rate depends on environmental temperature^17,18^ and varies markedly over short and long timescales. Since thermoregulation affects metabolism, it will also affect ROS production^19^, and, as a corollary, potentially telomere erosion^20–22^ with concomitant effects at the individual and population level^9,23^.

Given the potential causal chain, temperature – metabolism – ROS production – anti-oxidation - telomere erosion, ectotherm telomere dynamics should be particularly affected by climatic effects during the time of year when lizards are active and bask to elevate their body temperature^9,24–26^. More surprisingly are perhaps when ectotherms have longer telomeres under warm conditions (e.g., scincid lizards^22^;) or shorter telomeres under cool conditions (fish^24^;). This suggests that predictions between environmental thermal conditions and telomere dynamics may not be straightforward and thus our ability to predict the consequences of directional climate change on telomere dynamics may be compromised. *In vitro* studies show that the thermal stability of telomeric sequences differs depending on which cations (sodium or potassium) that stabilize the t-loop, and what particular guanine-quadruplex (G4) structure this has^27^. In cultured human cells telomeres erode as temperature increases, while telomerase seems relatively unaffected even at 42 °C^28^. Thus, the equivocal effects of environmental temperature may be explained by the interplay between different thermal reaction norms for telomere attrition (imposed by metabolic shifts) and telomerase-mediated addition of telomeric sequences and DNA repair systems of the telomere sequence.

As temperature shifts between seasons, it sets the scene for season-dependent telomere dynamics. In some endotherms that act as ectotherms in the sense of clearly differentiated seasonal activity and major metabolic shifts (i.e. torpor), studies have shown a relationship between hibernation and telomere lengths. Specifically, in small rodents telomere lengthening or attrition (or both) are arrested during torpor and hibernation^29,30^. Furthermore, in edible dormouse (*Glis glis*), hibernation also comes at a cost of increased telomere attrition during arousal^31^. The season-dependent telomere dynamic in response to dramatic metabolic changes with time of year in these small rodents raises the question whether similar dynamics play out in reptilian ectotherms.

During summer, reptiles experience a spike in metabolism on a daily basis, due to temperature shifts, which should result in oxidative stress when anti-oxidation is non-perfect^25,32–34^ and telomere erosion is expected to increase unless balanced by telomerase^35,36^. In contrast, in winter a reptile’s activity is low due to low temperature and thus metabolism and oxidative stress should be correspondingly lower^4,33^. Similarly, weather variation between years could result in variation in activity patterns and metabolic rate and thus potentially alter telomere dynamics. Annual and seasonal environmental effects on telomeres have not been explored in reptiles.

Using a sand lizard (*Lacerta agilis*) long-term data set from the Asketunnan peninsula in southern Sweden, we investigate how the temperature during winter (hibernating under a snow cover) and summer (while active and basking in the sun) affects subsequent telomere length. This species is a small (up to 20 g), oviparous, sexually dimorphic (males are green, females greyish-brown), ground-dwelling lizard^37–39^, that lives for ca. five years on average in Sweden (up to a maximum of ca. 15 years in southern parts of its range in central Europe). Males have an average home range of ca 1000 m^2^, about ten times the size of that of females^39^, can be easily resighted and recaptured^37–39^, and thus lend themselves very well for a study of this kind. Long-term studies investigating temperature effects on telomeres are crucial if we are to understand the potential longer term effects of directional climate change on telomere traits.

## Results

**Figure 1:**
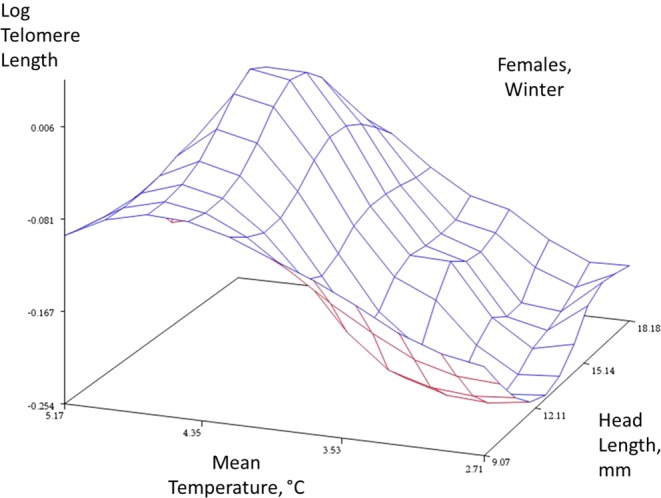
Winter data, females. Response surface plots with a spline function to describe the relationships between an index of age (head length, mm), mean temperature, and telomere length (log T/S ratio) in the two sexes (females, males) and two seasons (winter and summer). The spline function ‘smooths’ the plot to help reveal the complex relationship between these three traits (smoothing parameter = 0.5). The plot is descriptive and not directly associated with a statistical analysis.

**Figure 2:**
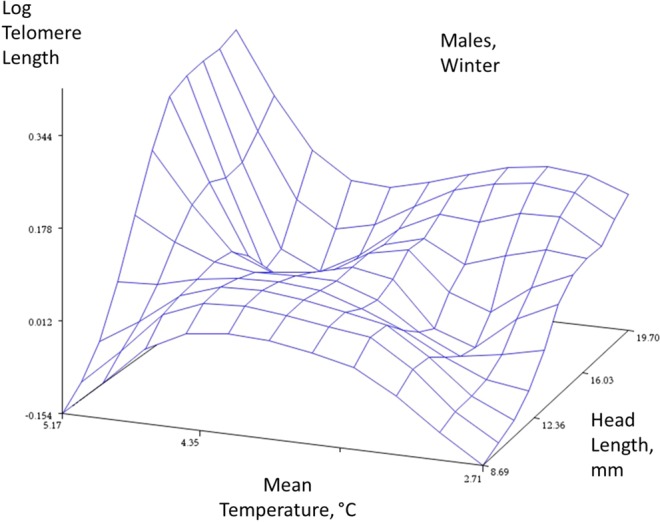
Winter data, males. Response surface plots with a spline function to describe the relationships between an index of age (head length, mm), mean temperature, and telomere length (log T/S ratio) in the two sexes (females, males) and two seasons (winter and summer). The spline function ‘smooths’ the plot to help reveal the complex relationship between these three traits (smoothing parameter = 0.5). The plot is descriptive and not directly associated with a statistical analysis.

Higher temperature in the summer preceding an individual’s telomere measurement was significantly and negatively related to telomere length, and so was the number of average sun hours in that period. (Table 1; Mean summer temperature = 15.2 °C ± 0.02, SE, n = 1037; Mean fraction of sunshine per hour (0–1) = 0.29 ± 0.0007, n = 1033; mean head length = 14.97 mm ± 0.07 mm). In winter, however, the results were reversed, with both mean temperature and sunshine being positively related to telomere length (Table 1; Mean winter temperature = 3.6 °C ± 0.02, SE, n = 1037; Mean fraction of sunshine per hour (0–1) = 0.12 ± 0.0006, SE; n = 1033; Mean head length = 14.97 mm ± 0.07 mm). The correlation between sunshine and mean temperature was negative and significant in winter (Spearman’s rank-order correlation, *r*_*s*_ = −0.11, *P < *0.0001, n = 1033), whereas in summer it was positive (*r*_*s*_ = 0.567, *P* < 0.0001, n = 1033). The interaction between head length and sex explained significant variance in telomere length in both seasons, but there was no significant effect of sex itself on telomere length with the interaction in the model. The ‘tilt’ and curvature of the response surfaces with respect to sex and season are captured in Figs. 1, 2 (winter) and 3, 4 (summer).

**Table 1:** Solution of fixed effects for telomere length in summer (a) and (b) winter.

a – Summer					
Effect	sex	Estimate	DF	T Value	Pr > |t|
Intercept		0.593 ± 0.098	808	6.04	<0.0001
Sex	F	−0.014 ± 0.068	808	−0.21	0.837
Sex	M		0	.	.
mmeanC		−0.019 ± 0.009	808	−2.22	0.027
Msunhour		−1.835 ± 0.376	808	−4.88	<0.0001
Head length		0.018 ± 0.003	808	5.84	<0.0001
GapdhRunNo		−0.00008 ± 0.00002	808	−1.62	0.106
Head length*sex	F	−0.012 ± 0.005	808	−2.60	0.009
Head length*sex	M	0		.	.
**b – Winter**					
Intercept		0.529 ± 0.063	808	−8.34	<0.0001
Sex	F	−0.043 ± 0.068	808	−0.63	0.529
Sex	M	0	.	.	.
mmeanC		0.076 ± 0.007	808	11.05	<0.0001
Msunhour		0.556 ± 0.229	808	2.43	0.015
Head length		0.015 ± 0.003	808	5.25	<0.0001
GapdhRunNo		−0.0001 ± 0.00005	808	−2.32	0.021
Head length*sex	F	−0.010 ± 0.004	808	−2.22	0.026
Head length*sex	M	0		.	.

The analysis is based on 556 individuals, 309 females and 247 males. Telomere plate number was backwards-eliminated in both analysis (P > 0.5; AIC was 16.8 units lower when removed). Individual ID is included as a random effect in all analyses to control for pseudoreplication arising from repeat observations of the same individual. Winter and summer analyses were modeled separately.

**Figure 3:**
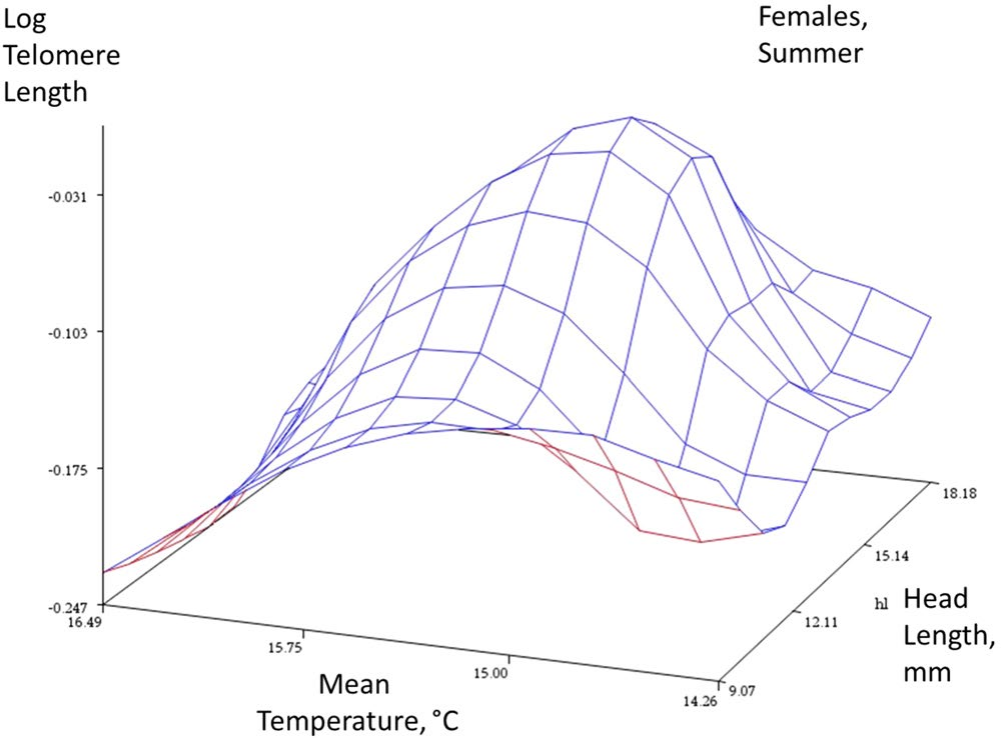
Summer data, females. Response surface plots with a spline function to describe the relationships between an index of age (head length, mm), mean temperature, and telomere length (log T/S ratio) in the two sexes (females, males) and two seasons (winter and summer). The spline function ‘smooths’ the plot to help reveal the complex relationship between these three traits (smoothing parameter = 0.5). The plot is descriptive and not directly associated with a statistical analysis.

**Figure 4:**
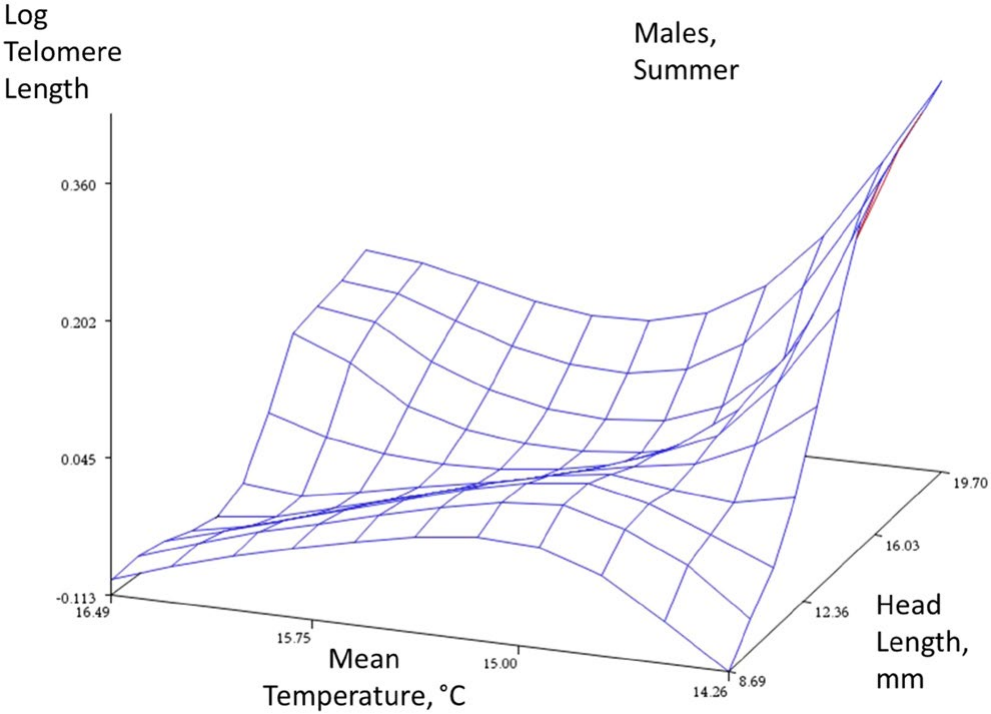
Summer data, males. Response surface plots with a spline function to describe the relationships between an index of age (head length, mm), mean temperature, and telomere length (log T/S ratio) in the two sexes (females, males) and two seasons (winter and summer). The spline function ‘smooths’ the plot to help reveal the complex relationship between these three traits (smoothing parameter = 0.5). The plot is descriptive and not directly associated with a statistical analysis.

## Discussion

Our analyses of sand lizards from over a decade-long field study in the wild show contrasting effects of environmental conditions on telomere dynamics during summer and winter; higher summer temperature and more sun were largely associated with shorter telomeres, whereas in winter, the opposite relationships applied – warmer conditions and more sun largely predicted longer telomeres. Although sun significantly affects telomere length in opposite ways in summer (−) and winter (+), the magnitude of the effect of sunshine is likely to be different in the two seasons. In summer, sunshine directly drives basking behaviour, whereas during hibernation, with the lizards hibernating in the ground and most often under snow cover, sun can only be expected to have a moderate effect via temperature. Thus it seems to agree with logic that the absolute parameter estimates (as a proxy for effect size) is nearly four times as high in summer (and negative) as the positive effect in winter (−1.84, *P* < 0.0001 vs. 0.56, *P* = 0.02).

Given the potential causal chain from temperature through metabolism and ROS production to telomere erosion, ectotherm telomere dynamics should be particularly affected by climatic effects during the time of year when lizards are active and bask to elevate their body temperature^9,17,33^. Our results thus agree with our prediction that a higher temperature during activity would result in telomere attrition and are in agreement with some, but not all, studies. For example, our results are concordant with a recent study on the desert agama (*Phrynocephalus przewalskii*) in which simulated heat waves in a laboratory resulted in the shortening of telomeres^26^. It also supports recent work on the European common lizard (*Lacerta vivipara*) in which population-specific reductions in telomere length in hatchlings were putatively linked with climate warming and inferred as an early warning sign of population extinction risk^23^. However, the picture is not straightforward and predictions of wide-scale effects of weather and climate patterns on telomere dynamics and long-term fitness consequences may be premature. Specifically, in other ectotherm species, warmer conditions can lead to longer telomeres as opposed to shorter ones as we found, although in a non-linear relationship. In the Tasmanian snow skink (*Niveoscincus ocellatus*), warmer conditions led to a significant increase in telomere length during the typical length of an activity season^22^. Similarly, in the Eastern mosquitofish (*Gambusia holbrooki*), higher temperature during growth was associated with reduced telomere attrition^24^. The explanation for these disparate results likely lies in the dynamics of telomere regulation. Telomere length is a net result of erosion (i.e., cellular division and oxidative stress^3,4^;), those that regulate oxidative stress (anti-oxidants) and those that lengthen telomeres (predominantly telomerase^1^;). If these processes have different thermal optima and show different (perhaps non-linear) ‘dose-response’ relationships with temperature (e.g.^28,40^), this could explain some of the seasonal thermal effects we report here.

Given the reduced metabolic rate in winter, and potentially, oxidative stress, the prediction of reduced telomere attrition in winter in this ectotherm is straightforward, however, explaining a largely positive relationship between winter temperature and telomere length is more complex^41,42^. Typically, torpor and hibernation are considered a mechanism to reduce metabolic expenditure and as a result, in endotherms, typically slows telomere attrition. For example, in bears (*Ursus americanus*) shortened hibernation periods are associated with accelerated telomere attrition^43^ and in the edible dormouse (*Glis glis*) hibernation halts telomere attrition^30^. In Djungarian hamsters (*Phodopus sungorus*) daily torpor also slows telomere attrition but does so in a sex-specific manner; higher temperature during torpor (20 °C versus 9 °C) had less effect on slowing attrition^29^. These effects are thus in the opposite direction to ours, suggesting species-specific effects.

Effects of environmental conditions on rates of telomere attrition are expected to be especially pronounced in ectotherms (as we have shown here) because the strong relationship between environmental condition, activity, metabolic rate and this potentially oxidative-stress effects on telomeres. However, environmental effects are not restricted to ectotherms and have been increasingly demonstrated in a range of taxa. For example, in free-living, black-tailed gulls (*Larus crassirostris*) telomeric stability or lengthening were associated with years of benign environmental conditions and high food availability, while decreases in telomere length were characterized by reduced food availability^44^. In Seychelle’s warblers (*Acrocephalus sechellensis*) telomere length was positively associated with temporal variation in environmental conditions (island-wide insect abundance)^45^. Environmental effects also play out over larger scales; in stone chats (*Saxicola* spp), Aspfelbach *et al*.^46^ provide evidence that distinct selective pressures in tropical and temperate environments may be reflected in divergent patterns of telomere loss with more favorable environmental conditions potentially minimising telomere shortening. While there are emerging patterns in birds, studies in mammals are scarcer. In a rare exception, Wilbourn *et al*.^47^ showed that telomeres were shorter in roe deer (*Capreolus capreolus*) populations experiencing poor environmental conditions, but these effects were only apparent in older individuals and resulted from a significant increase in telomere length in older animals under benign conditions.

For both our summer and winter data sets, our combined proxy of age and size (i.e., head length^37^) had a positive relationship with telomere length, but this effect is sex-specific with a weaker relationship in females. Importantly, head length is correlated with age also when body size is controlled for in both sexes^37^. In addition, relative head size is larger in males and telomere length appears to be weakly positively correlated with age in female, but not male, sand lizards (62). We report elsewhere^48^ that in a limited data set with repeat-observations of telomere length through life, there was a significant interaction effect between sex and real age on telomere length. Thus, this supports the analyses in the current MS with a significant interaction between the age proxy (head length) and sex on telomere length in a much larger data set. In our previous analysis, the main effect of age on telomere length was non-significant^48^, whereas in the current analysis there is a significant head length-sex interaction, which motivates careful interpretation of the main effects of sex and the age proxy *per se* (Table 1). In Proc Mixed SAS 9.4, the presence of a Type III main effect is tested after the other main effect and their interaction^49^, which here casts doubt on the main effect of head length and its interaction with sex. It can also not be ignored that the different head length – body size relations in the two sexes may add non-linear scaling effects that complicates interpretation of this sex-dependent age proxy effect on telomere length.

There was thus no independent effect of sex on telomere length and the effect sizes overall were weak. This differs to some extent from a previous study of ours in which females had marginally longer age-corrected telomeres than males^38^. Several factors may contribute to this difference in outcome: (i) The current analysis contains some four times as many individuals, many sampled repeatedly (controlled for as a random effect). Thus, the analyses are based on different samples. (ii) Head length in relation to body size and age does not show identical trajectories in the two sexes^39^ and, hence, head length cannot be expected to show the exact same relationship with telomere length in the two sexes. However, here this is irrelevant since we only want to control for any size and age effects that may confound our analysis of seasonal thermal effects on variance in telomere length. (iii) In the previous analysis^38^, we used terminal restriction fragment analysis (TRF), whereas in the current analysis we used qPCR. To what extent this makes a difference in analytical outcomes is hard to evaluate, with qPCR showing greater variance than TRF in some studies^50,51^, but not others^52^, while TRF has a greater risk of missing smaller telomeres^53,54^. Although these differences in results should be noted, they have no impact on the analyses of temperature and seasonality effects on telomere length. Our results from both these studies contradict the paradigm from many endotherms that age and telomere length should covary – potentially highlighting the continued activity of telomerase throughout life in somatic tissue^9,10^ but the results from the current study suggest these effects are manifested primarily through the period surrounding winter rather than summer during which temperature had negative effects on telomere length.

## Conclusion

Understanding telomere dynamics in free-living populations is crucial to understanding their role in evolutionary and ecological processes, especially their role in mediating life-history trade-offs. Increasingly the role of environmental effects on telomeres are being appreciated because telomere length represents a promising biomarker of overall physiological state and of past environmental experiences, which could help us understand the drivers of life-history variation in natural populations with the potential to contributing to our understanding of climate change impacts in individual and population fitness. Our study is one of few to both contrast seasonal effects and examine the effects in free-living ectotherms. A conclusion from our opposing effects in summer and winter is that telomere dynamics in the wild are complex and potentially predicting downstream effects on long-term climate change may not be straightforward. Warm environmental conditions during summer was probably associated with telomere attrition but these largely negative effects could be balanced if winter conditions are warmer than usual. This has implications for ectotherm life histories, and potentially for telomere-mediated selection for choice of optimal hibernation sites in winter and basking regimes in summer. Further work is required to understand these dynamics and to appreciate the fitness consequences in this and other natural systems. Hypotheses generated by our results should be testable using already collected data in many cases, such as blood samples in already parentage-assigned populations sampled for DNA, in combination with climatological data, and experimentally verifiable using factorial-design thermal experiments in captivity.

## Methods

### Model system

Climatological data was obtained from the Swedish Bureau of Meteorology and Hydrology (SMHI) for the relevant time period 1997–2005. This data was retrieved from the nearest climate data logger, located in Varberg, ca 50 km south of our lizard field site at Asketunnan. Both these localities are situated on the coast and at the same elevation and can be considered equivalent in terms of cloud cover, rainfall and sunshine (i.e., basking opportunities in summer). Thus, data collected should reflect corresponding annual weather variation at the study site^55–57^. From a sampling perspective, data on climatological traits were collected in year x, and their relations to telomere traits and morphometry measured in year (x + 1) as outlined below.

The data on *Lacerta agilis* used for this study was collected during the years 1998–2006. Methods of the field procedures, including sampling of whole blood from *vena angularis* (nicked with the tip of a needle in the corner of the mouth with the blood collected in a capillary tube, transferred to 70% alcohol, kept on ice, and frozen to −80 C for long term storage). The procedures follow well established protocols that have been described in detail elsewhere^38,45,55–57^. In brief, the study was carried out in Southern Sweden at our Asketunnan study population (57°22N, 11°59′E). In each year, the population was monitored intensively during their active season with repeat observations of individual lizards within and among years. Lizards were caught by noose or hand and all blood sampling and recording of morphometrics took place in spring (April-June, at first capture in the season, mostly in early May) in the year after the weather had been recorded; thus, the underlying logic here is that we want to assess any effects on telomeres from previous summer and winter weather conditions. Detailed hibernation conditions is poorly described in sand lizards but one male sand lizard was exposed during the clearing of an egg laying site in the mid 1980’s only ca 15 cm under *Calluna* heath and ca 30 cm snow cover (Olsson pers. obs.). Thus, depending on overwintering sites, lizards are likely to be exposed to thermal fluctuations buffered by snow insulation in the dark.

### Telomere length measurements: qPCR

DNA was extracted from blood samples using the Qiagen Puregene blood kit (Cat No. 158467). Telomere length was measured using quantitative polymerase chain reaction (qPCR) since this method is efficient and can handle large sample sizes^58^. All qPCR work followed^59^ and^60^ with slight modifications. To evaluate DNA concentration and purity, we used a PHERAstar F5 Spectrophotometer (BMG Labtech, Germany). The total yield averaged 527 ng/µl per sample, with high molecular purity (mean A260/280 = 1.76; n = 2476). Samples with low yield and/or low quality were excluded, other samples were diluted to a working concentration of 20 ng/µl. The limit for exclusion was set to 5 ng/ µl and 1.4 for all our analyses but these applied only to juvenile samples. DNA concentrations ranged from 5.96 ng/ µl to 1090.61 ng/ µl. All samples were diluted to 20 ng/ul, and nine samples that were less than 20 ng/ µl, were not diluted and the number of microliters of DNA added was increased to total approximately 20 ng/ µl. The A260/280 range was 1.4–1.95.

Previously published primers^60^ were used to amplify the control single copy gene glyceraldehydes-3-phosphate dehydrogenase (GAPDH) and telomere primers (Telb1 and Telb2). A standard curve was generated for GAPDH and telomere primers using six five-fold serial dilutions of DNA from an arbitrary *L. agilis* sample to generate a reference curve. Each run included a positive control, no template control and the same reference standard to control for the qPCR’s amplification efficiency and set up to the threshold Ct value; every sample was run in triplicate. For maximized precision, if a triplicate had a standard deviation >0.3 it was rerun. The DNA concentrations at each point of the standard curve was: (1) 500 ng (log template DNA = 2.7)), (2) 100 ng (log template DNA = 2.0), (3) 20 ng (log template DNA = 1.3, (4) 4 ng (log template DNA = 0.6), (5) 0.8 ng (log template DNA = −0.1), (6) 0.16 ng (log template DNA = −0.8).

All PCR analyses were performed on a RotorGene6000 (Qiagen, Australia). Using the product recommended PCR conditions, 11.25 µl SensiMix SYBR no-ROX Master Mix (Bioline, Australia) was included in the 20 µl final volume of both 200 nM concentration primer sets. 1 ul DNA per well was added at a concentration of 20 ng/ul, except for 9 samples that yielded less than 20 ng/ul. Up to 2–4 ul was added for each of those samples.

Non-specific products were not amplified, indicated by a single peak in the melt curve analysis for each reaction. To analyse baseline fluorescence, individual efficiencies and window of linearity per amplicon, LinRegPCR 12.18 was used^61,62^. The cycle at which the fluorescence level crosses the threshold, which is proportional to the quantity of DNA in a sample, is represented by the threshold cycle values (C_t_). For each sample, for both genes, C_t_ values were obtained. Each sample’s telomere length (T) was expressed relative to the single gene control (S; GAPDH). The Pfaffl method^63^ was used to calculate relative telomere length (T/S ratio) as the deviation in qPCR reading from a reference DNA sample (GAPDH), i.e. relative telomere to single copy (T/S) ratio (adapted from^59^). Inter-assay coefficient of variation (mean ± STD) for qPCR runs for telomere (n = 123) and GAPDH (n = 176) amplification were 3.85 ± 0.59% and 7.73 ± 1.87%, respectively. Mean amplification efficiency across all qPCR runs (n = 299) was 1.999 ± 0.013 (STD).

### Statistical analysis

Data on sunshine was available from SMHI as number of absolute minutes of sun in a given hour around the clock. We divided the number of sun minutes by 60 to derive the fraction of ‘sunshine hours’ for any given hour. Temperatures (max-, min- and mean-) and rainfall were averaged to daily means. All climatological data was then averaged to grand means per season to match the single observation of telomere length per individual and year. Winter (inactive period) was defined as October – April, and summer (active period) as May – September. Linear mixed modelling was performed in Statistical Analysis Software (SAS) 9.4 to assess inter-annual variation in telomere length, followed by corresponding covariation of telomere length (T/S ratio) with each climate parameter, head length, and sex as fixed effects, and individual ID as a random factor using Proc Mixed in SAS 9.4. Head length (mm) was used as a combined representation for age and body size to maximize the sample size, since real age was not known for all individuals (in *L. agilis* there is a strong correlation between age and head size^37^). The same 556 individuals (309 females, and 247 males) were used repeatedly in the summer and winter analysis. To adjust for the repeated measures, all mixed model procedures were run with Kenward-Roger correction to calculate degrees of freedom and control for type I error. For the final model, and to avoid correlations between predictors, the three temperature grand means were averaged to a mean temperature. The outcomes did not change qualitatively between full and reduced models. Telomere plate number did not explain any variation in summer or winter analyses (p > 0.78 for both analyses, the AIC value was 16.8 units lower when removed from the analysis), whereas there was a slight effect of GAPDH run number in winter (p = 0.026, Table 1), and we therefore kept GAPDH run number in both final models.

### Ethics approval and consent to participate

All work conforms to Swedish animal welfare and conservation legal requirements as approved by the Animal Ethics Committee at the University of Gothenburg, Sweden (Ethics Permit No. 73-2014).

## Data Availability

The dataset supporting the conclusions of this article is available in the [repository name] repository, [unique persistent identifier and hyperlink to dataset(s) in http:// format].”
